# Electronic Medical Record–Based Machine Learning Approach to Predict the Risk of 30-Day Adverse Cardiac Events After Invasive Coronary Treatment: Machine Learning Model Development and Validation

**DOI:** 10.2196/26801

**Published:** 2022-05-11

**Authors:** Osung Kwon, Wonjun Na, Heejun Kang, Tae Joon Jun, Jihoon Kweon, Gyung-Min Park, YongHyun Cho, Cinyoung Hur, Jungwoo Chae, Do-Yoon Kang, Pil Hyung Lee, Jung-Min Ahn, Duk-Woo Park, Soo-Jin Kang, Seung-Whan Lee, Cheol Whan Lee, Seong-Wook Park, Seung-Jung Park, Dong Hyun Yang, Young-Hak Kim

**Affiliations:** 1 Division of Cardiology Department of Internal Medicine, Eunpyeong St Mary's Hospital Catholic University of Korea Seoul Republic of Korea; 2 Department of Medical Science, Asan Medical Institute of Convergence Science and Technology, Asan Medical Center University of Ulsan College of Medicine Seoul Republic of Korea; 3 Division of Cardiology, Department of Internal Medicine, Asan Medical Center University of Ulsan College of Medicine Seoul Republic of Korea; 4 Division of Cardiology, Department of Internal Medicine, Ulsan University Hospital University of Ulsan College of Medicine Ulsan Republic of Korea; 5 Artificial Intelligence Lab, Linewalks, Inc Seoul Republic of Korea; 6 Department of Radiology, Asan Medical Center University of Ulsan College of Medicine Seoul Republic of Korea

**Keywords:** big data, electronic medical record, machine learning, mortality, adverse cardiac event, coronary artery disease, prediction

## Abstract

**Background:**

Although there is a growing interest in prediction models based on electronic medical records (EMRs) to identify patients at risk of adverse cardiac events following invasive coronary treatment, robust models fully utilizing EMR data are limited.

**Objective:**

We aimed to develop and validate machine learning (ML) models by using diverse fields of EMR to predict the risk of 30-day adverse cardiac events after percutaneous intervention or bypass surgery.

**Methods:**

EMR data of 5,184,565 records of 16,793 patients at a quaternary hospital between 2006 and 2016 were categorized into static basic (eg, demographics), dynamic time-series (eg, laboratory values), and cardiac-specific data (eg, coronary angiography). The data were randomly split into training, tuning, and testing sets in a ratio of 3:1:1. Each model was evaluated with 5-fold cross-validation and with an external EMR-based cohort at a tertiary hospital. Logistic regression (LR), random forest (RF), gradient boosting machine (GBM), and feedforward neural network (FNN) algorithms were applied. The primary outcome was 30-day mortality following invasive treatment.

**Results:**

GBM showed the best performance with area under the receiver operating characteristic curve (AUROC) of 0.99; RF had a similar AUROC of 0.98. AUROCs of FNN and LR were 0.96 and 0.93, respectively. GBM had the highest area under the precision-recall curve (AUPRC) of 0.80, and the AUPRCs of RF, LR, and FNN were 0.73, 0.68, and 0.63, respectively. All models showed low Brier scores of <0.1 as well as highly fitted calibration plots, indicating a good fit of the ML-based models. On external validation, the GBM model demonstrated maximal performance with an AUROC of 0.90, while FNN had an AUROC of 0.85. The AUROCs of LR and RF were slightly lower at 0.80 and 0.79, respectively. The AUPRCs of GBM, LR, and FNN were similar at 0.47, 0.43, and 0.41, respectively, while that of RF was lower at 0.33. Among the categories in the GBM model, time-series dynamic data demonstrated a high AUROC of >0.95, contributing majorly to the excellent results.

**Conclusions:**

Exploiting the diverse fields of the EMR data set, the ML-based 30-day adverse cardiac event prediction models demonstrated outstanding results, and the applied framework could be generalized for various health care prediction models.

## Introduction

Cardiovascular disease is the leading cause of mortality throughout the world and is associated with various morbidities [1]. Invasive treatment, including percutaneous coronary intervention (PCI) and coronary artery bypass grafting (CABG) surgery, is commonly required in patients with acute coronary syndrome and stable angina. Owing to the potential risk associated with inevitable invasiveness and the individual comorbidities, risk stratification and identification of high-risk patients is warranted [2,3]. Accordingly, several risk prediction models for adverse events after invasive coronary treatment have been proposed [4-7]. However, their use is limited owing to inadequate predictive ability, low generalizability, and lack of individualized risk assessment, as they have been developed using limited number of variables in select cohorts.

In recent times, with an increase in the availability of large volume of electronic medical record (EMR) data, there has been a gradual interest in using data-driven approaches to construct efficient tools for risk prediction [8,9]. In addition, machine learning (ML) algorithms are gaining popularity as an alternative approach for risk prediction to deal with complex EMR data and to overcome the limitations of previous models [10]. Recent work on models based on EMR data for predicting adverse events suggests that incorporation of ML might allow more accurate risk prediction [11-14]. However, validated robust models are still limited, as the previous models used prespecified variables based on traditional risk factors mainly comprising structural data or lacked proper external validation. Thus, this study aimed to develop ML models by utilizing diverse fields of both structured and unstructured EMR data to predict the risk of 30-day major adverse cardiac events (MACE), including mortality, after PCI or CABG and to validate the model in a different cohort.

## Methods

### Database

#### Development and Internal Validation Set

The data for this study were obtained from Asan Medical Center, which provides quaternary medical care for people in South Korea. It has 55 departments—approximately 2700 beds—and >8000 employees; it sees approximately 3,000,000 outpatient clinic visits and 900,000 admissions per year. The Asan biomedical research environment is the data warehouse system of Asan Medical Center, which has deidentified information of 4 million patients and is updated every 3 days [15]. The Asan heart registry was constructed from diverse fields of structured or unstructured EMR data extracted from the Asan biomedical research environment database by using structured query language. The registry comprised 571,157 patients, and the inclusion criteria were inpatient admissions or outpatient visits in the cardiology, cardiac surgery, or emergency department for established or suspected heart diseases between January 1, 2000 and November 30, 2016.

#### External Validation Set

For external validation, we used data obtained from the EMRs of Ulsan University Hospital, which is a tertiary hospital with approximately 900 beds that caters to a metropolitan city and its surrounding suburban area in the southern region of South Korea. The patients’ demographics, medical practice, and operating systems differ between the 2 hospitals, which would allow evaluation of the model in a different population.

### Data Processing

**Figure 1:**
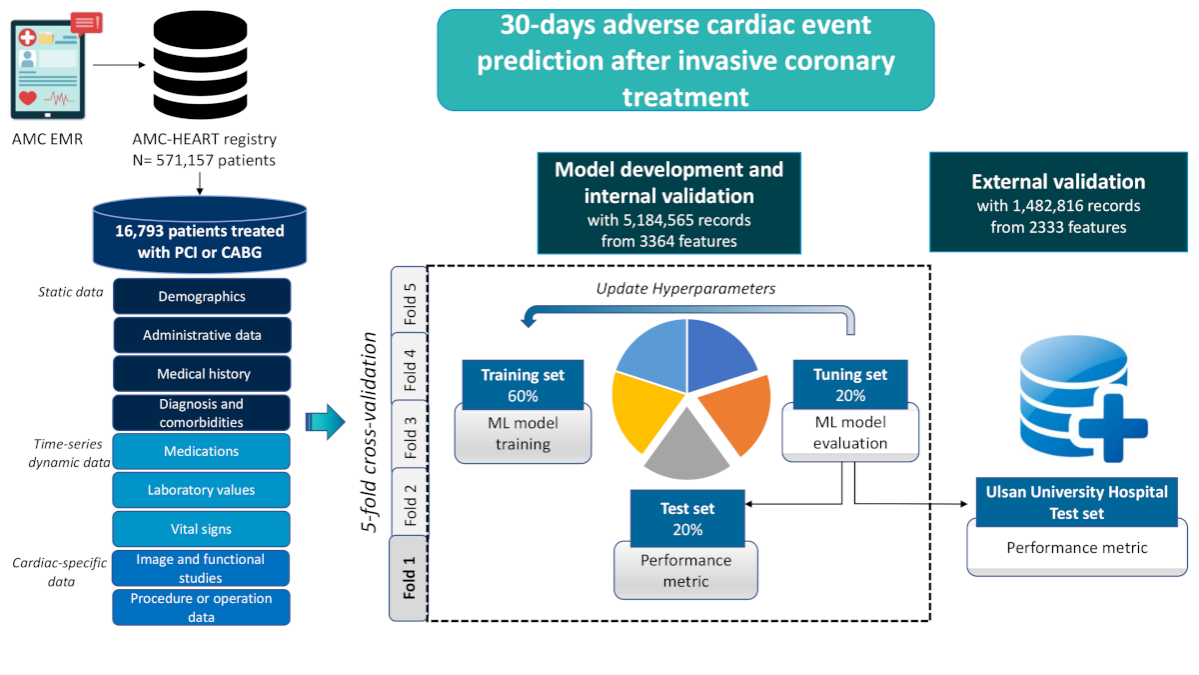
Study diagram. Database, machine learning, and validation. AMC: Asan Medical Center; CABG: coronary artery bypass grafting; EMR: electronic medical record; ML: machine learning; PCI: percutaneous coronary intervention.

The overall process for building the EMR-based database is presented in Figure S1 of Multimedia Appendix 1. Briefly, first, we collected the anonymized records of 748,474 patients who had visited the Asan Medical Center or Ulsan University Hospital because of cardiovascular diseases. Second, we set clinically plausible criteria to remove errors and duplications. Third, we integrated unstructured data such as readings of medical examinations with structured data sourced from EMRs to create the CardioNet [16]. We subsequently performed text mining to structuralize the significant variables associated with cardiovascular diseases because most results of the principal cardiovascular diseases–related medical examinations are free-text readings. The basic method of text mining applied to unstructured data can be described in 3 steps. First, we created a metatable consisting of the main variables and conditions of extraction by the clinician. Second, we divided the readings into 3 frames: text, tabular, and others, and defined the extraction rules for each frame. We took into consideration the structure of the original data and the location of variables set in the metatable and defined rules by using a variety of operators and regular expressions. Third, the new tables were built by extracting the keywords and features from the original data. The values of the keywords were based on rules defined in the previous step. Additionally, to ensure interoperability for convergent multicenter research, we standardized the data by using several codes that correspond to the common data model. Finally, we created the descriptive table (ie, dictionary of the CardioNet) to simplify access and utilization of data for clinicians and engineers and continuously validated the data to ensure reliability [16]. Most structured data were obtained using classic preprocessing technologies, including data cleansing, data integration, data transformation, data reduction, and privacy protection. Finally, we extracted the following structured data elements: demographics, administrative information, medical history and comorbidities, diagnoses, vital signs, laboratory values, and medications. Unstructured data included the following elements: reports of cardiac-specific studies such as thallium-201 single-photon emission computed tomography (SPECT), coronary angiography, and physicians’ procedure notes for PCI or CABG. In this study, we found that with the algorithms developed, we classified the data into 3 categories: basic static data (demographics, administration data, medical history, comorbidities, and diagnosis), dynamic time-series data (medications, laboratory values, and vital signs), and disease-specific data (electrocardiography, treadmill test, echocardiography, coronary computerized tomography, thallium-201 SPECT, coronary angiography, PCI, and CABG) (see Figure 1). The details of the variables in each category are presented in Table S1 in Multimedia Appendix 1 [16]. With respect to the data of the procedures or operation, the variables only confined to the index PCI or CABG were used for this investigation. Data collection and preparation were approved by the Asan Medical Center and Ulsan University Hospital institutional review board, and the requirement for informed consent was waived. Patient deidentification was performed in line with the Health Insurance Portability and Accountability Act. This report adheres to the transparent reporting of a multivariable prediction model for individual prognosis or diagnosis reporting guideline [17].

### Study Population and Outcome

**Figure 2:**
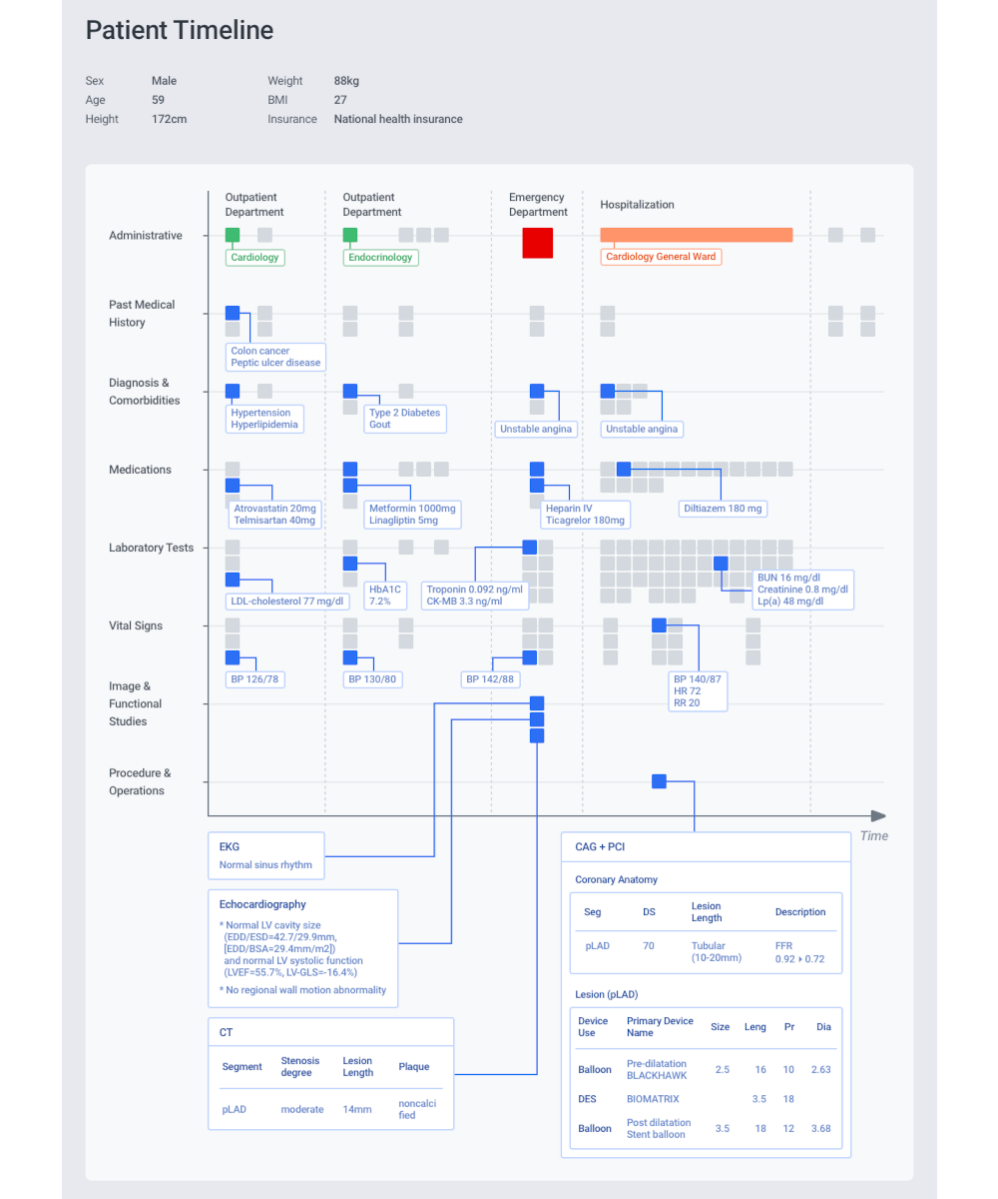
An example case incorporating serial and various electronic medical record data to predict adverse events. BP: blood pressure; BSA: body surface area; BUN: blood urea nitrogen; CAG: coronary angiography; CK-MB: creatine kinase myocardial band; Dia: diameter; EDD: end diastolic dimension; EF: ejection fraction; EKG: electrocardiogram; ESD: end systolic dimension; FFR: fractional flow rate; GLS: global longitudinal strain; Hb: hemoglobin; HR: heart rate; LDL: low-density lipoprotein; Leng: length; Lp(a): lipoprotein A; LV: left ventricle; PCI: percutaneous coronary intervention; pLAD: proximal left anterior descending; Pr: pressure; RR: respiratory rate.

A cohort of 16,793 patients who had undergone PCI (n=12,519) or CABG (n=4274) between January 1, 2006 and November 30, 2016 was identified in the Asan heart registry. As the majority of patients underwent the index PCI or CABG within 1 year after their first generation of data in EMR, we fairly used 1-year accumulated data prior to index procedures for the entire population. The total number of independent records in the data set was 5,184,565, derived from 3364 features. Figure 2 illustrates an example of the patients treated with PCI, encompassing the serial and various EMR data. In the external validation cohort from Ulsan University Hospital, 4159 patients comprising 3950 who underwent PCI and 209 who underwent CABG between January 1, 2006 and November 30, 2016 were included. The data set consisted of 1,482,816 records from 2333 features. Mortality was the primary endpoint, captured through documentation of mortality in the EMR based upon National Health Insurance information. MACE as the secondary endpoint referred to a composite of all-cause mortality, including myocardial infarction, stroke, or repeat revascularization at 30 days following the index invasive treatment. Myocardial infarction, stroke, and repeat revascularization were initially identified from source documents, including diagnosis, electrocardiography, laboratory tests, procedural notes, and results of imaging studies such as magnetic resonance imaging or computerized tomography. Subsequently, the events were rigorously adjudicated by cardiologists or neurologists according to the current definitions [18].

### ML Algorithms and Statistics

We only used data generated until index PCI or CABG, whereas data obtained after the index procedure were excluded for developing ML algorithms (see Figure 2). Three approaches were applied to preprocess data generated until index procedures: (1) history-aware encoding is used to reflect whether clinical events had occurred before a certain period of time, (2) one-hot encoding is used to express the existence and missingness of variables, and (3) characteristics of time-series variables were captured by using descriptive statistics (eg, minimum, maximum, average, and count). The detailed explanation regarding time-series data analysis is shown in Multimedia Appendix 2. The study population was randomly split into training, tuning, and validation cohorts in a ratio of 3:1:1. Four commonly used classes of ML algorithms were used: logistic regression (LR), random forest (RF), gradient boosting machine (GBM), and feedforward neural network (FNN). LR transforms output by using a logistic sigmoid function. RF is an extension of the bagging method, as it utilizes both bagging and feature randomness to create an uncorrelated forest of decision trees and decide output by majority voting using multiple decision trees. GBM is similar to RF, except that they build 1 tree at a time and combine the voting results in a gradual, additive, and sequential manner. FNN is a classic type of deep learning model that uses hierarchical layers of abstraction and computes the output by using a combination of multiple nodes with nonlinear activation.

The hyperparameters for each model were determined using an empirical search and 5-fold cross-validation on the study population to determine the values that had the best performance (see Figure 1). Hyperparameters and their values in each model are summarized in Table 1. The optimal values of the tuning parameters were identified based on the testing accuracy values that were calculated for each fold and averaged. External validation of the developed prediction models was performed in a cohort from a different hospital. In addition, we determined the performance of each data category and checked the cumulative performance with combinations of multiple information categories, adding each category one by one to identify the best performance. Development of risk algorithms in the training cohort and application of the risk algorithms to the validation cohort was completed using Python with library packages “Keras with Tensorflow backend.” To investigate the important variables in each developed model, we used the permutation feature importance algorithm for LR and FNN, Gini impurity for RF, and frequency of variables for GBM.

**Table 1:** Hyperparameters and those values of each model.

Model, hyperparameter		Value
**Logistic regression**		
	Solver	liblinear
	Maximal iteration	100
**Random forest**		
	Number of estimators	100
	Maximal depth	10
**Gradient boosting machine**		
	Objective	binary
	Estimators	150
	Boosting type	Gradient boosting decision tree
	Number of leaves	15
	Maximal depth	–1 (no limit)
	Learning rate	0.025
	Minimal number of data in child	90
**Feedforward neural network**		
	Learning rate	0.0002
	Hidden layer units	(64,64)
	Batch size	64
	Epoch	40
	Dropout rate	0.5
	Optimizer	Adam (beta1=.5, beta2=.999)

The descriptive characteristics of the study population are provided as number (%) and mean (SD) for categorical and continuous variables, respectively. The discrimination performance of each model was evaluated based on the area under the receiver operating characteristic curve (AUROC) and area under the precision-recall curve (AUPRC). In addition, we evaluated model calibration (ie, the model’s ability to accurately predict the observed absolute risk) by using the Brier score, where 0 would indicate perfect calibration, and generated the calibration plots. A 2-sided *P* value <.05 was considered indicative of statistical significance. We did not perform any imputation of the missing numerical values, as explicit imputation of missing values does not always provide consistent improvements in predictive models based on electronic health records [19,20]. Because of inevitable differences in the characteristics and amounts of data between cohorts, we used binary indicators of missingness on external validation [21].

## Results

### Baseline Characteristics and Event Rates

The baseline characteristics of the population in the development and internal validation groups are listed in Table 2. The mean patient age was 62.7 (SD 10.2) years; of the 16,793 patients, 12,465 (74.2%) were males and 6084 (36.2%) had diabetes, while 243 (1.4%) had a history of congestive heart failure. Chronic renal insufficiency, chronic lung disease, and chronic liver disease were reported in 566 (3.4%), 386 (2.3%), and 487 (2.9%) patients, respectively. Approximately two-thirds of the patients were admitted via outpatient clinics while the remaining patients were admitted via the emergency department. Among 16,793 patients in our developmental cohort, MACE at 30 days occurred in 1500 (8.9%) patients, including 178 cases (1.1%) of mortality, 1159 (6.9%) cases of myocardial infarction, 124 (7.4%) cases of stroke, and 180 (1.1%) cases of repeat revascularization. Among a total of 4159 patients in the external validation cohort, there were 75 (1.8%) mortalities at 30 days follow-up; the details of the patients’ characteristics in the external validation cohort are shown in Table 3.

**Table 2:** Baseline clinical characteristics of the development and internal validation set.

Characteristics	Development and internal validation set		
	Total population (N=16,793)	Percutaneous coronary intervention (n=12,519)	Coronary artery bypass grafting surgery (n=4274)
Age (years), mean (SD)	62.7 (10.2)	62.2 (10.5)	64.1 (9.4)
Male sex, n (%)	12,465 (74.2)	9312 (74.4)	3153 (73.8)
Body mass index (kg/m^2^), mean (SD)	24.9 (3.1)	25.0 (3.0)	24.6 (3.1)
Hypertension, n (%)	10,697 (63.7)	7758 (62)	2939 (68.8)
Diabetes mellitus, n (%)	6084 (36.2)	4127 (33)	1957 (45.8)
Hyperlipidemia, n (%)	9200 (54.8)	6932 (55.4)	2268 (53.1)
Current cigarette smoker, n (%)	3009 (17.9)	2424 (19.4)	585 (13.7)
Prior myocardial infarction, n (%)	568 (3.4)	394 (3.1)	174 (4.1)
Previous cerebrovascular accident, n (%)	596 (3.5)	420 (3.4)	176 (4.1)
History of congestive heart failure, n (%)	243 (1.4)	132 (1.1)	111 (2.6)
Peripheral vascular disease, n (%)	278 (1.7)	199 (1.6)	79 (1.8)
Valvular heart disease, n (%)	387 (2.3)	106 (0.8)	281 (6.6)
Chronic renal insufficiency, n (%)	566 (3.4)	363 (2.9)	203 (4.7)
Chronic lung disease, n (%)	386 (2.3)	306 (2.4)	80 (1.9)
Chronic liver disease, n (%)	487 (2.9)	396 (3.2)	91 (2.1)
History of malignancy, n (%)	1019 (6.1)	816 (6.5)	203 (4.7)
Presentation with acute myocardial infarction, n (%)	3032 (18.1)	2509 (20)	523 (12.2)
Admission via emergency department, n (%)	5054 (30.1)	3941 (31.5)	1113 (26)
Admission via outpatient clinics, n (%)	11,739 (69.9)	8578 (68.5)	3161 (74)

**Table 3:** Baseline clinical characteristics of the external validation set.

Characteristics	External validation set		
	Total population (n=4159)	Percutaneous coronary intervention (n=3950)	Coronary artery bypass grafting surgery (n=209)
Age (years), mean (SD)	61.7 (10.9)	61.6 (9.4)	62.7 (10.9)
Male sex, n (%)	2913 (70)	2779 (70.3)	134 (64.1)
Body mass index (kg/m^2^), mean (SD)	24.0 (5.4)	24.0 (5.2)	23.8 (6.4)
Hypertension, n (%)	1947 (46.8)	1851 (46.8)	96 (45.9)
Diabetes mellitus, n (%)	1278 (30.7)	1195 (30.2)	83 (39.7)
Hyperlipidemia, n (%)	1154 (27.7)	1098 (27.7)	56 (26.7)
Current cigarette smoker, n (%)	1285 (30.9)	1234 (31.2)	51 (24.4)
Prior myocardial infarction, n (%)	280 (6.7)	265 (6.7)	15 (7.1)
Previous cerebrovascular accident, n (%)	233 (5.6)	220 (5.5)	13 (6.2)
History of congestive heart failure, n (%)	76 (1.8)	71 (1.7)	5 (2.3)
Peripheral vascular disease, n (%)	49 (1.1)	45 (1.1)	4 (1.9)
Valvular heart disease, n (%)	27 (0.6)	18 (0.4)	9 (4.3)
Chronic renal insufficiency, n (%)	130 (3.1)	123 (3.1)	7 (3.3)
Chronic lung disease, n (%)	146 (3.5)	143 (3.6)	3 (1.4)
Chronic liver disease, n (%)	201 (4.8)	193 (4.8)	8 (3.8)
History of malignancy, n (%)	192 (4.6)	183 (4.6)	9 (4.3)
Presentation with acute myocardial infarction, n (%)	1357 (32.6)	1314 (33.2)	43 (20.5)
Admission via emergency department, n (%)	1706 (41)	1634 (41.3)	72 (34.4)
Admission via outpatient clinics, n (%)	2453 (58.9)	2316 (58.6)	137 (65.5)

### Performance in Predicting 30-Day Mortality

**Figure 3:**
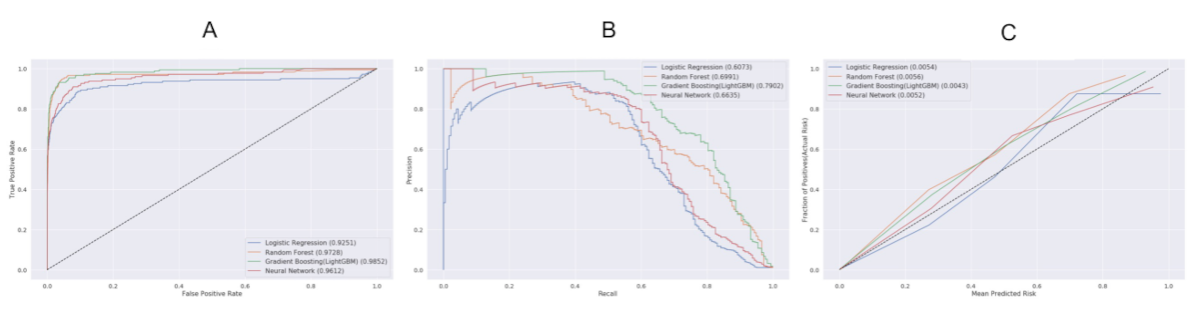
Five-fold cross-validation of performance of each machine model in predicting 30-day mortality after invasive treatment. A. Area under the receiver-operator characteristic curve, B. Area under the precision-recall curve, and C. Calibration plot with Brier score.

Figure 3 demonstrates the discrimination and calibration results of 5-fold cross-validation obtained by evaluation with each technique. GBM showed the highest AUROC with a value of 0.99 (95% CI 0.97-0.99, *P*<.001) and RF showed similar AUROC of 0.98 (95% CI 0.96-0.0.99, *P*<.001) (see Figure 3A). The AUROCs of FNN and LR were slightly lower at 0.96 (95% CI 0.93-0.99, *P*<.001) and 0.93 (95% CI 0.87-0.99, *P*<.001), respectively. GBM had the highest AUPRC with a value of 0.80, and AUPRCs of RF, LR, and FNN were 0.73, 0.68, and 0.63, respectively (see Figure 3B). In terms of model calibration, all models showed low Brier scores of less than 0.1, indicating an excellent fit of the ML-based models (see Figure 3C). Calibration plots for each model also confirmed good agreement between the estimated predicted risk and observed risk.

**Figure 4:**
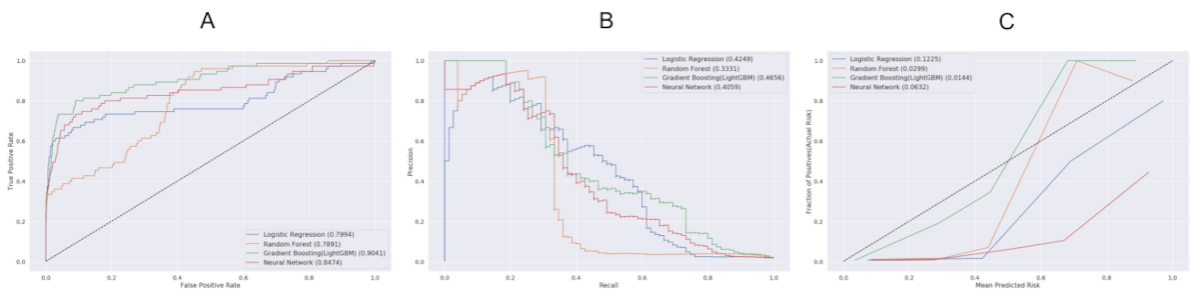
External validation of performance of each machine model in predicting 30-day mortality after invasive treatment. A. Area under the receiver operator characteristic curve, B. Area under the precision-recall curve, and C. Calibration plot with Brier score.

On external validation using the data set of the Ulsan University hospital, maximal predictive performance was observed with GBM (AUROC 0.90, 95% CI 0.86-0.95; *P*<.001), followed by FNN with AUROC of 0.85 (95% CI 0.81-0.92, *P*<.001) (see Figure 4A). LR and RF showed slightly lower AUROCs of 0.80 (95% CI 0.73-0.87, *P*<.001) and 0.79 (95% CI 0.74-0.84, *P*<.001), respectively. The AUPRCs of GBM, LR, and FNN showed similar values of 0.47, 0.42, and 0.41, respectively; however, that of RF was lower at 0.33 (see Figure 4B). All models showed low Brier scores of <0.1, indicating a good fit of the ML-based models (see Figure 4C).

**Figure 5:**
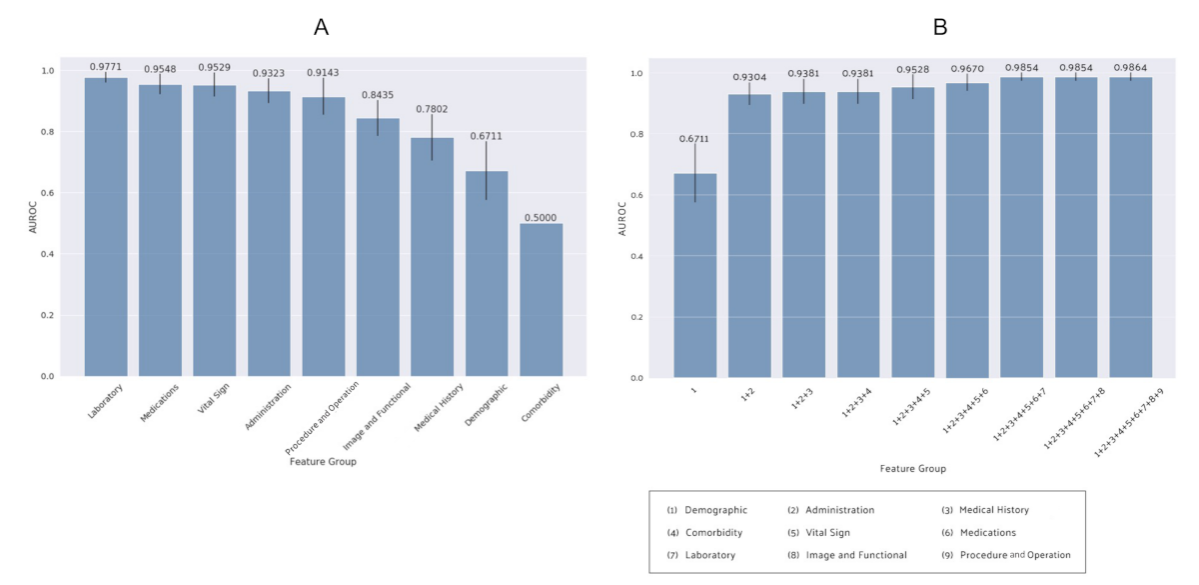
Prediction performance of the gradient boosting machine model assessed by area under the receiver operator characteristic curves. A. Each data category, B. Combination of data categories. AUROC: area under the receiver operator characteristic curve.

Figure 5A illustrates the predictive performance of each data category in GBM, which showed the highest AUROC. Among the individual categories, laboratory values demonstrated the highest AUROC with a value of 0.98. Medications and vital signs showed the second highest AUROCs with a value of 0.95. In contrast, static data such as diagnosis and comorbidities category, data, and medical history showed low AUROCs of <0.80. GBM using combinations of feature categories showed progressive improvement in performance, while dynamic time-series data was gradually included on top of the basic static data, after which subtle improvement was seen when adding cardiac-specific data (see Figure 5B).

### Performance in Predicting MACE

The performance of the ML models for predicting 30-day MACE is demonstrated in Table 4. The maximal predictive performance was observed with GBM (AUROC 0.88, 95% CI 0.85-0.90; *P*<.001). RF and FNN showed a similar performance with AUROCs of 0.85 (95% CI 0.83-0.88, *P*<.001) and 0.85 (95% CI 0.83-0.88, *P*<.001), respectively, while the AUROC of the LR was lower at 0.83 (95% CI 0.82-0.88, *P*<.001). In terms of the AUPRC, GBM showed the highest value of 0.50, followed by FNN, RF, and LR with values of 0.41, 0.39, and 0.37, respectively. All models showed low Brier scores of less than 0.1, indicating a good fit of the ML-based models.

**Table 4:** Performance of machine learning models for predicting major adverse cardiac events.

Model	Area under the receiver operating characteristic curve	95% CI	*P* value	Area under the precision-recall curve	Brier score
Logistic regression	0.83	0.82-0.88	<.001	0.37	0.06
Random forest	0.85	0.83-0.88	<.001	0.39	0.06
Gradient boosting machine	0.88	0.85-0.90	<.001	0.50	0.05
Feedforward neural network	0.85	0.83-0.88	<.001	0.41	0.06

### Calculating the Importance of Feature Variables in Mortality-Prediction Models

The rank of important variables in the models for predicting 30-day mortality is presented in Table 5. In LR, systolic blood pressure was identified as the most important variable. RF indicated serum aspartate aminotransferase as important, while GBM and FNN indicated serum protein and serum phosphorus important, respectively. Overall, vital signs and several laboratory values such as arterial blood pH, O_2_, and CO_2_ concentration were mainly identified as important variables across the different ML methods.

**Table 5:** Top 10 important variables of each machine learning model.

Rank	Logistic regression	Random forest	Gradient boosting machine	Feedforward neural network
1	Systolic blood pressure	Serum aspartate aminotransferase	Serum protein	Serum phosphorus
2	Diastolic blood pressure	Pa_CO2_	Age	Pa_CO2_
3	Respiratory rate	Arterial pH	Serum phosphorus	Hemoglobin
4	Pa_CO2_	Pa_O2_	Systolic blood pressure	Systolic blood pressure
5	Arterial pH	Serum alanine aminotransferase	Platelet	Normal sinus rhythm in electrocardiogram
6	Pa_O2_	Total bilirubin	Serum aspartate aminotransferase	Estimated glomerular filtration rate
7	Aspartate aminotransferase	Creatine kinase-myocardial band	Pa_O2_	Serum glucose
8	Pulse rate	White blood cell	Serum albumin	Platelet
9	Blood urea nitrogen	Serum sodium	Pulse rate	Pa_O2_
10	Serum phosphorus	Platelet	Activated partial thromboplastin time	Arterial pH

## Discussion

### Principal Findings

This was a retrospective study that applied ML to structured and unstructured patient data from the EMR of a large quaternary hospital to develop a risk prediction model for 30-day adverse cardiac events in patients who underwent PCI or CABG. We comparatively evaluated the performance of several models; all models demonstrated outstanding results with AUROCs more than 0.90 with excellent calibration. On external validation, the performance in predicting 30-day mortality decreased; however, it remained favorable. Dynamic time-series data, including laboratory values, vital signs, and medications, demonstrated the best performances, which mainly contributed to outstanding performance of the models.

Traditional risk prediction models are derived from a small set of selected risk factors based on the significant univariate relationship with the end point on LR, which might deteriorate the predictive performance. Moreover, it is difficult to include new and more discriminatory risk factors into the traditional models, which limits their extension ability [12]. Advances in big data solutions allow for storage, management, and mining of large volumes of structured and semistructured data such as complex health care data [22]. Along the emergence of big data, ML provides an alternative approach to establish prediction modeling that might address the current limitations. In this context, we aimed to develop and validate ML models by using longitudinal and heterogeneous data of various EMR parameters to predict mortality or MACE at 30 days after PCI or CABG. In addition, we explored a general framework for constructing models by categorizing the data set into static basic data, dynamic time-series data, and disease-specific data to examine the potential applicability. This study revealed encouraging results, which indicate that ML-based models for predicting adverse events after invasive coronary treatment might be feasible and applicable as a clinical decision supporting system in hospitals with fully implemented EMR protocols. Furthermore, this approach can be extended to various disease entities or clinical events for improvement in quality of care and patient outcomes.

In this study, we found that the algorithms developed from a large single-center EMR database were reliable for use in the population of a different hospital, albeit with a relatively low performance. Of note, different hospitals serve dissimilar patient populations and have divergent clinical practice patterns; therefore, the EMR data reflecting the real-world clinical practice in each hospital has its own distinct characteristics. Hence, a somewhat low performance of the proposed prediction models in a different cohort can be anticipated. Ideally, a model that achieves the highest possible level of generalizability is desirable. However, there have been concerns about whether a model developed at 1 center can be applied to another center [9]. In medicine, there are too many practice patterns and other local idiosyncrasies that make learning a broadly applicable model effectively difficult [23,24]. In respect that the ultimate application of prediction models built with EMR data is integration with the clinical decision support system for personalized medicine, optimizing individual centers’ particular prediction model may be more important rather than extending generalizability. Hence, although the developed algorithms from a single-center EMR database can be used with the database of a different hospital, individual prediction models based on the EMR data of each single hospital would be preferable for highly optimized performance.

Predictive models with EMR data frequently rely on structured data. However, given the volume and richness of data available in unstructured clinical notes or reports, ML models might benefit from leveraging text mining tools to enhance the model [22,25]. Hence, we text-mined various cardiac-specific data such as image and functional studies and detailed information about PCI and CABG, although the process required diverse strategies and tasks. In this study, text-mined cardiac-specific data showed a fair ability to predict 30-day mortality risk. Although valuable, there are still some challenges in applying text-mined data in ML, particularly owing to the vagueness, impreciseness, and uncertain clinical information in EMR data [12]. In contrast, utilizing structured data is simple if the database and automated process system for extraction, transformation, and loading of data are well-established. Algorithms with only time-series dynamic data, which is the typical large-volume structured data, outperformed and primarily contributed to the excellent final results. Intuitively, it is believed that the learning model will perform better if more data are integrated into learning [26]. Our results indicate that using only large amount of reliable structured data of EMR could offer an opportunity to develop proper risk prediction models. However, although improvement in clinical data collection processes is necessary, fundamentally, significant clinical information should be recorded digitally in a cohesive and standardized manner in the EMR system.

### Limitations

Several limitations of this study should be noted. First, the cardiovascular event rates, including mortality, might be underestimated because events were captured only from a single-center EMR database. Linking it with the national claim data or health insurance data might possibly capture the events more accurately. Second, although ease of interpretation is vital for evaluation of the models [27,28], the black box nature of ML makes it difficult to be used in health care. Hence, we tried to assess the importance of the variables through several experiments; however, there is still a lack of “explainability” of the prediction models. For ML methods to be readily adopted in real-world clinical practice, they must be interpretable without compromising on accuracy [29]. Future works focusing on developing explainable ML models are necessary to provide tailored feedback to physicians. Third, other ML methods such as recurrent neural networks, which have shown advantage in leveraging the dynamic features, were not investigated in this work; this needs to be explored in future studies [26,29]. Fourth, although EMR data within 1 year before index procedures were used for all populations, different EMR follow-up times prior to index procedures were not taken into account to develop models. Finally, we did not conduct external validation for MACE. Because physician adjudication of myocardial infarction, stroke, or repeat revascularization events is resource-intensive and time-consuming in a large-scaled record cohort, comprehensive source reviews and final ascertainment were substantially challenged. In order to expand the use of the EMR-based ML approach, optimization for computerized detection and adjudication of clinical outcomes will require considerable investment of time and collaboration with institutional information technology and bioinformatics professionals.

### Conclusion

Exploiting the diverse parameters of EMR data sets, we developed and validated ML models for predicting the 30-day mortality risk following PCI or CABG. The ML algorithms showed excellent performance, and the applied framework can be generalized for various health care prediction models. This study suggests that ML using the real-word clinical data set can provide a substantial method of developing risk prediction models. Future studies are warranted to establish the clinical effectiveness of this approach and real-time application at the point of care.

## Appendix

Multimedia Appendix 1 Supplementary figures and tables.

Multimedia Appendix 2 Time-series analysis.

## Abbreviations

AUPRC: area under the precision-recall curve
AUROC: area under the receiver operating characteristic curve
CABG: coronary artery bypass grafting
EMR: electronic medical record
FNN: feedforward neural network
GBM: gradient boosting machine
LR: logistic regression
MACE: major adverse cardiac events
ML: machine learning
PCI: percutaneous coronary intervention
RF: random forest
SPECT: single-photon emission computed tomography
