# Supplementary material for: Electronic Medical Record–Based Machine Learning Approach to Predict the Risk of 30-Day Adverse Cardiac Events After Invasive Coronary Treatment: Machine Learning Model Development and Validation
Source: JMIR Med Inform. 2022 May 11;10(5):e26801. doi: 10.2196/26801 (PMC9133980; doi:10.2196/26801)
Supplement: Multimedia Appendix 1 [file medinform_v10i5e26801_app1.docx]

**Multimedia Appendix 1**

Kwon O. and Na W. et al. “Electronic Medical Record–Based Machine Learning Approach to Predict the Risk of 30-Day Adverse Cardiac Events After Invasive Coronary Treatment: Machine Learning Model Development and Validation”

**Figure S1. The overall process for building the EMR-based database**

**Table S1. Data categories and detail variables used in developing machine learning models.**

This supplementary material was provided by the authors to provide readers with additional information of their work.

**Figure S1. The overall process for building the EMR-based database [16].**

There are a total of five steps involved: data extraction, structuralization, cleansing, standardization, and validation.


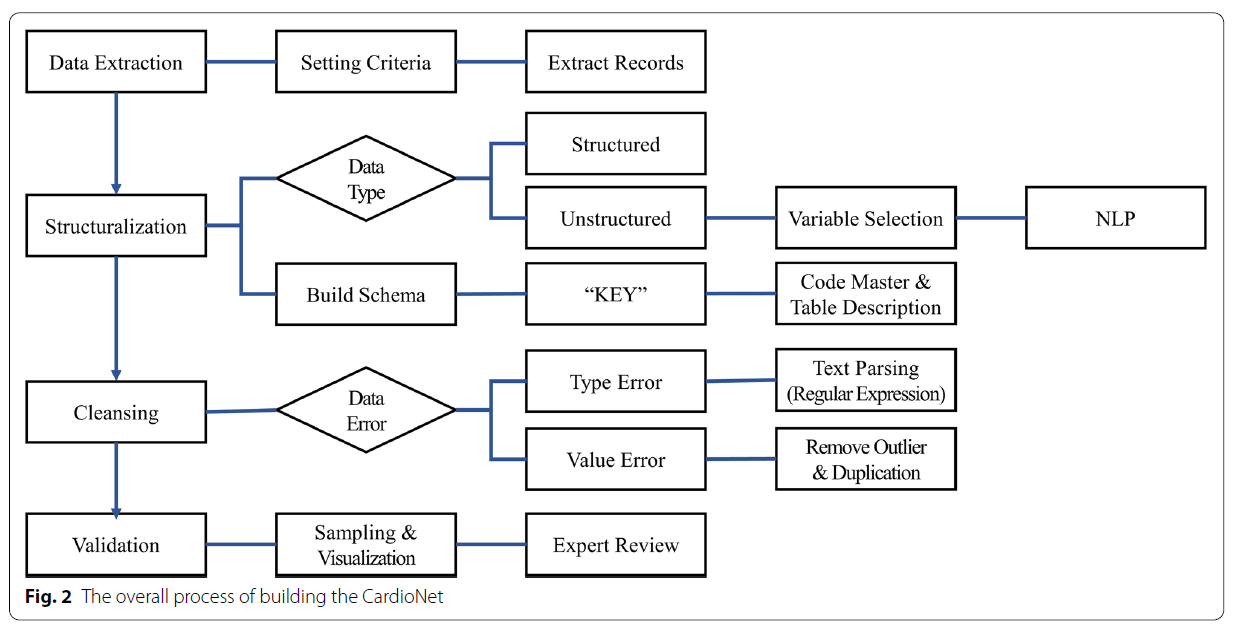
The details on how to establish the EMR-based database of the Asan heart registry was demonstrated in the individual article titled “CardioNet: A manually curated database for artificial intelligence-based research on cardiovascular diseases” by Ahn et al.

**Table S1. Data Categories and Detail Variables Used in Development and Validation of Machine Learning Models**

| **Basic static data** | **Development and internal validation set**  **from Asan Medical Center** | **External validation set**  **from Ulsan University Hospital** |
| --- | --- | --- |
| Demographics | Height  Weight  Body mass index  Age  Sex  Race  Insurance  Marital status  Level of education  Religion | Height  Weight  Body mass index  Age  Sex  Race |
| Administrative data | Duration of hospitalization  Hospitalization within the past year  Presentation to emergency department  Service department (cardiac surgery, cardiology, non-cardiac surgery, or cardiology)  Number of prior inpatient admission within the past year  Prior outpatient visits  Stay at the intensive care unit  Length of intensive care unit stay | Duration of hospitalization  Hospitalization within the past year  Presentation to emergency department  Service department (cardiac surgery, cardiology, non-cardiac surgery, or cardiology)  Number of prior inpatient admission within the past year  Prior outpatient visits  Stay at the intensive care unit |
| Past medical history | Diabetes  Insulin-dependent diabetes  Hypertension  Hyperlipidemia  Current smoking  Alcohol drinking  Previous myocardial infarction  Ischemic heart disease  Prior cardiac surgery  Prior percutaneous coronary intervention  Prior coronary artery bypass grafting surgery  Congestive heart failure  Other cardiomyopathy  Valvular heart disease  Atrial fibrillation and/or flutter  Peripheral vascular disease  Chronic renal dysfunction  Dialysis  Anemia  Transient ischemic attack  Stroke  Hypothyroidism  Hyperthyroidism  Chronic liver disease  Pulmonary circulation disorder  Chronic lung disease  Lymphoma/leukemia  Solid cancer  Connective tissue disorder  Rheumatoid arthritis  Depression disorder  Anxiety disorder  Delirium  Psychoses  Dementia  Other neurologic disorders  Decubitus ulcer  Fracture  Peptic ulcer disease | Diabetes  Insulin-dependent diabetes  Hypertension  Hyperlipidemia  Current smoking  Previous myocardial infarction  Ischemic heart disease  Prior cardiac surgery  Prior percutaneous coronary intervention  Prior coronary artery bypass grafting surgery  Congestive heart failure  Other cardiomyopathy  Valvular heart disease  Atrial fibrillation and/or flutter  Peripheral vascular disease  Chronic renal dysfunction  Dialysis  Transient ischemic attack  Stroke  Hypothyroidism  Hyperthyroidism  Chronic liver disease  Pulmonary circulation disorder  Chronic lung disease  Lymphoma/leukemia  Solid cancer  Connective tissue disorder  Rheumatoid arthritis  Depression disorder  Anxiety disorder  Delirium  Psychoses  Dementia  Other neurologic disorders  Decubitus ulcer  Fracture  Peptic ulcer disease |
| Diagnosis | ST-elevation myocardial infarction  Non-ST-elevation myocardial infarction  Unstable angina  Stable angina  Certain current complications following acute myocardial infarction  Congestive heart failure  Other cardiomyopathy  Valvular heart disease  Ventricular tachycardia/fibrillation  Atrial fibrillation/Flutter  Renal failure  Dialysis  Fluid and electrolyte disorder (Hyperkalemia, hypokalemia, hypernatremia, hyponatremia)  Hypothyroidism  Hyperthyroidism  Acute pneumonia  Sepsis  Shock  Transient ischemic attack  Stroke  Chronic liver disease  Pulmonary circulation disorder  Chronic lung disease  Lymphoma/leukemia  Solid cancer  Connective tissue disease  Rheumatoid arthritis  Depression disorder  Anxiety disorder  Delirium  Psychoses  Dementia  Other neurologic disorder(s)  Decubitus ulcer  Fracture  Peptic ulcer disease | ST-elevation myocardial infarction  Non-ST-elevation myocardial infarction  Unstable angina  Stable angina  Certain current complications following acute myocardial infarction  Congestive heart failure  Other cardiomyopathy  Valvular heart disease  Ventricular tachycardia/fibrillation  Atrial fibrillation/Flutter  Renal failure  Dialysis  Fluid and electrolyte disorder (Hyperkalemia, hypokalemia, hypernatremia, hyponatremia)  Hypothyroidism  Hyperthyroidism  Acute pneumonia  Sepsis  Shock  Transient ischemic attack  Stroke  Chronic liver disease  Pulmonary circulation disorder  Chronic lung disease  Lymphoma/leukemia  Solid cancer  Connective tissue disease  Rheumatoid arthritis  Depression disorder  Anxiety disorder  Delirium  Psychoses  Dementia  Other neurologic disorder(s)  Decubitus ulcer  Fracture  Peptic ulcer disease |
| **Dynamic time-series data** |  |  |
| Medications | Insulin  Statin  Other lipid-lowering agent(s)  Omega-3 fatty acid supplement  P2Y12 inhibitors  Aspirin  Non-steroidal anti-inflammatory drugs  Immunosuppressant  Beta-blocker  Angiotensin-converting enzyme inhibitors  Angiotensin receptor blocker  Calcium-channel blocker  Hydralazine  Antiarrhythmics  Digoxin  Aldosterone antagonist  Warfarin  Novel oral anticoagulants  Other anticoagulant(s)  Loop diuretics  Thiazide  Nitrates  Intravenous vasopressor or inotropics  Medications for diabetes  Antibiotics  Steroid  Red blood cell transfusion  Platelet transfusion  Fresh frozen plasma transfusion  Cryoprecipitate transfusion | Insulin  Statin  Other lipid-lowering agent(s)  Omega-3 fatty acid supplement  P2Y12 inhibitors  Aspirin  Non-steroidal anti-inflammatory drugs  Immunosuppressant  Beta-blocker  Angiotensin-converting enzyme inhibitors  Angiotensin receptor blocker  Calcium-channel blocker  Hydralazine  Antiarrhythmics  Aldosterone antagonist  Novel oral anticoagulants  Other anticoagulant(s)  Loop diuretics  Thiazide  Nitrates  Intravenous vasopressor or inotropics  Medications for diabetes  Antibiotics  Steroid  Red blood cell transfusion  Platelet transfusion  Fresh frozen plasma transfusion  Cryoprecipitate transfusion |
| Laboratory values | Hemoglobin  White blood cell  Band cells  Platelet  Erythrocyte sedimentation rate  C-reactive protein  High sensitivity-C-reactive protein  B-type natriuretic peptide  Pro- B-type natriuretic peptide  Troponin-I  Creatine kinase-myocardial band  Creatine kinase  Creatinine  Blood urea nitrogen  Estimated glomerular filtration rate  Protein  Albumin  Alkaline phosphatase  Total bilirubin  Aspartate transaminase  Alanine aminotransferase  Arterial pH  Arterial pO_2_  Arterial pCO_2_  Arterial HCO_3_  International normalized ratio of prothrombin time  Activated partial thromboplastin time  fibrinogen  Fibrinogen degradation production  Antithrombin III  D-dimer  Ferritin  HbA1c  Plasma glucose  Apoliprotein B-100  Apoliprotein A-1  Lipoprotein (a)  Homocysteine  Uric acid  Total cholesterol  Triglyceride  High density lipoprotein-cholesterol  Low density lipoprotein -cholesterol  Sodium  Potassium  Chloride  Calcium  Phosphorus  Thyroid-stimulating hormone  Free T4 | Hemoglobin  White blood cell  Band cells  Platelet  Erythrocyte sedimentation rate  C-reactive protein  High sensitivity-C-reactive protein  B-type natriuretic peptide  Troponin-I  Creatine kinase-myocardial band  Creatine kinase  Creatinine  Blood urea nitrogen  Estimated glomerular filtration rate  Protein  Albumin  Alkaline phosphatase  Total bilirubin  Aspartate transaminase  Alanine aminotransferase  Arterial pH  Arterial pO_2_  Arterial pCO_2_  Arterial HCO_3_  International normalized ratio of prothrombin time  Activated partial thromboplastin time  fibrinogen  Fibrinogen degradation production  Antithrombin III  D-dimer  Ferritin  HbA1c  Plasma glucose  Apoliprotein B-100  Apoliprotein A-1  Lipoprotein (a)  Homocysteine  Uric acid  Total cholesterol  Triglyceride  High density lipoprotein-cholesterol  Low density lipoprotein -cholesterol  Sodium  Potassium  Chloride  Calcium  Phosphorus  Thyroid-stimulating hormone  Free T4 |
| Vital signs | Systolic blood pressure  Diastolic blood pressure  Heart rate  Respiratory rate  Temperature | Systolic blood pressure  Diastolic blood pressure  Heart rate  Respiratory rate  Temperature |
| **Cardiac-specific data (image and functional studies)** |  |  |
| Electrocardiography | Sinus rhythm  Mobitz II block  Complete heart block  Atrial fibrillation/flutter  Pacemaker rhythm  Supraventricular tachycardia  Junctional rhythm  Wide QRS tachycardia | Sinus rhythm |
| Treadmill | Negative/Equivocal/Positive |  |
| Echocardiography | Left ventricular systolic dimension  Left ventricular diastolic dimension  Left ventricular posterial wall thickness at the systolic phase  Left ventricular posterial wall thickness at the diastolic phase  Left ventricular septal thickness at the systolic phase  Left ventricular septal thickness at the diastolic phase  Left atrial size  Inner diameter of the aorta  Right ventricular diameter at the level of the outflow tract at the systolic phase  Right ventricular diameter at the level of the outflow tract at the diastolic phase  Mitral valve peak E velocity  Mitral valve peak A velocity  Ratio of E/A  Mitral valve deceleration time  Mitral valve area measured by 2D  Mitral valve area measured by pressure half time  Maximal pressure gradient of the mitral valve  Mean pressure gradient of the mitral valve  Degree of mitral valve regurgitation  Jet area of mitral valve regurgitation  Left ventricular outflow tract diameter  Maximal velocity at the left ventricular outflow tract  Mean velocity at the left ventricular outflow tract  Aortic valve area measured by 2D  Degree of aortic valve regurgitation  Degree of tricuspid valve regurgitation  Maximal velocity of tricuspid valve regurgitation  Pressure gradient between the right ventricle and atrium  Degree of pulmonary valve regurgitation  Peak velocity of pulmonary flow  Main pulmonary artery diameter  Location of regional wall motion abnormality  Degree of regional wall motion abnormality  Wall motion index  Pericardial effusion at the anterial space of the left ventricle  Pericardial effusion at the posterial space of the left ventricle  Left ventricular mass index  Tissue Doppler image E at the septum  Tissue Doppler image A at the septum  Mitral valve E/E’ ratio  Proximal isovelocity surface area at the mitral valve  Pressure gradient at the left ventricular outflow tract  Aortic valve area by Doppler  Sinus diameter  Sinotubular junction diameter  Tubular diameter  Jet area of tricuspid valve regurgitation  Peak velocity of pulmonary valve regurgitation  Velocity of pulmonary valve regurgitation at the diastolic phase  Maximal pressure gradient of pulmonary valve  Mean pressure gradient of pulmonary valve  Left ventricular ejection fraction  Left ventricular mass  Time-velocity integral at the aortic valve  Tissue Doppler image E at the lateral side  Tissue Doppler image A at the lateral side  Time-velocity integral at the level of the left ventricular outflow tract  Pulmonary flow at the systolic phase  Pulmonary flow at the diastolic phase | Left ventricular systolic dimension  Left ventricular diastolic dimension  Left ventricular posterial wall thickness at the systolic phase  Left ventricular posterial wall thickness at the diastolic phase  Left ventricular septal thickness at the systolic phase  Left ventricular septal thickness at the diastolic phase  Left atrial size  Inner diameter of the aorta  Right ventricular diameter at the level of the outflow tract at the systolic phase  Right ventricular diameter at the level of the outflow tract at the diastolic phase  Mitral valve peak E velocity  Mitral valve peak A velocity  Ratio of E/A  Mitral valve deceleration time  Mitral valve area measured by 2D  Mitral valve area measured by pressure half time  Maximal pressure gradient of the mitral valve  Mean pressure gradient of the mitral valve  Degree of mitral valve regurgitation  Jet area of mitral valve regurgitation  Left ventricular outflow tract diameter  Maximal velocity at the left ventricular outflow tract  Mean velocity at the left ventricular outflow tract  Aortic valve area measured by 2D  Degree of aortic valve regurgitation  Degree of tricuspid valve regurgitation  Maximal velocity of tricuspid valve regurgitation  Pressure gradient between the right ventricle and atrium  Degree of pulmonary valve regurgitation  Peak velocity of pulmonary flow  Main pulmonary artery diameter  Location of regional wall motion abnormality  Degree of regional wall motion abnormality  Wall motion index  Pericardial effusion at the anterial space of the left ventricle  Pericardial effusion at the posterial space of the left ventricle  Left ventricular mass index  Tissue Doppler image E at the septum  Tissue Doppler image A at the septum  Mitral valve E/E’ ratio  Proximal isovelocity surface area at the mitral valve  Pressure gradient at the left ventricular outflow tract  Aortic valve area by Doppler  Sinus diameter  Sinotubular junction diameter  Tubular diameter  Jet area of tricuspid valve regurgitation  Peak velocity of pulmonary valve regurgitation  Velocity of pulmonary valve regurgitation at the diastolic phase  Maximal pressure gradient of pulmonary valve  Mean pressure gradient of pulmonary valve  Left ventricular ejection fraction  Left ventricular mass  Time-velocity integral at the aortic valve  Tissue Doppler image E at the lateral side  Tissue Doppler image A at the lateral side  Time-velocity integral at the level of the left ventricular outflow tract  Pulmonary flow at the systolic phase  Pulmonary flow at the diastolic phase |
| Thallium-SPECT | Abnormality of Bull’s eye  Location of perfusion defect  Severity of perfusion defect  Size of perfusion defect |  |
| Coronary angiography | Coronary Anatomy  Lesion  Degree of stenosis  Lesion-Type  Patent  Total/Subtotal occlusion  Chronic total occlusion  Fractional flow rate  Intracoronary ultrasound  Rupture  Thrombus  Tandem  Aneurysm  Bifurcation  Calcification  Diminutive  Dissection  In-stent restenosis  Jailed  Milking  Ulceration  Eccentric | Coronary Anatomy  Lesion  Degree of stenosis |
| Coronary computed tomography | Occlusion of native coronary artery  (vessel, location and degree)  Plaque composition  In-stent restenosis  Graft patency |  |
| **Cardiac-specific data (procedure and operation)** |  |  |
| Percutaneous coronary intervention | Vessel with lesions  Proximal part involvement  Pre-stent balloon  Post-stent balloon  Drug-eluting balloon  Cutting balloon  Bare-metal stent  Drug-eluting stent  Bioabsorbable stent  Direct atherectomy  Rotablation  Thrombectomy  Bail-out stenting  Kissing balloon  Balloon size  Balloon length  Balloon pressure  Maximal balloon size  Stent size  Stent length  Stent pressure  Maximal stent balloon size | Vessel with lesions  Pre-stent balloon  Post-stent balloon  Drug-eluting balloon  Bare-metal stent  Drug-eluting stent |
| Coronary artery grafting surgery | On-pump or off-pump  Number of arterial grafts  Number of vein grafts  Davinci operation | On-pump or off-pump  Number of arterial grafts  Number of vein grafts |
