# Supplementary material for: Electronic Medical Record–Based Machine Learning Approach to Predict the Risk of 30-Day Adverse Cardiac Events After Invasive Coronary Treatment: Machine Learning Model Development and Validation
Source: JMIR Med Inform. 2022 May 11;10(5):e26801. doi: 10.2196/26801 (PMC9133980; doi:10.2196/26801)
Supplement: Multimedia Appendix 2 [file medinform_v10i5e26801_app2.docx]

**Multimedia Appendix 2**

Kwon O. and Na W. et al. “Electronic Medical Record–Based Machine Learning Approach to Predict the Risk of 30-Day Adverse Cardiac Events After Invasive Coronary Treatment: Machine Learning Model Development and Validation”

**Time series analysis**

1) History-aware encoding using feature engineering for irregular time series

We used autocorrelation, stationarity, entropy and methods from the physic-based nonlinear time-series analysis literature because of diverse range of temporal patterns of the clinical information [*arXiv:1709.08055v2*].

2) One-hot encoding

We have examined the various methods to express the categorical variables effectively, and one-hot encoding which improved the performance was applied to express the existence and missingness of variables [*arXiv:1602.03686].*

3) Time-series statistics

We have pre-processed the time-series data by generating the statistical values for the specific time period using a tabular data augmentation method [*arXiv:2002.12478*].

**References**

1. arXiv:1709.08055v2; Feature-based time-series analysis

2. arXiv:1602.03686; Medical Concept Representation Learning from Electronic Health Records and its Application on Heart Failure Prediction

3. arXiv:2002.12478; Time Series Data Augmentation for Deep Learning: A Survey

**Table S1. Example of time-series statistics**

| Date time | Serum protein |
| --- | --- |
| 2014-12-02 09:25:00 | 7.0 g/dL |
| 2014-12-09 08:55:00 | 6.6 g/dL |
| 2014-12-16 09:00:00 | 5.9 g/dL |
| 2015-01-30 08:05:00 | 6.4 g/dL |
| 2015-02-13 08:40:00 | 5.8 g/dL |
| 2015-03-30 09:25:00 | 6.0 g/dL |
| 2015-03-31 03:40:00 | 4.8 g/dL |

| Look-back period | Frequency | Mean | Minimal value | Maximal value |
| --- | --- | --- | --- | --- |
| 1 week | 2 | 5.4 | 4.8 | 6.0 |
| 2 weeks | 2 | 5.4 | 4.8 | 6.0 |
| 1 month | 2 | 5.4 | 4.8 | 6.0 |
| 2 months | 4 | 5.75 | 4.8 | 6.4 |
| 3 months | 4 | 5.75 | 4.8 | 6.4 |
| 6 months | 7 | 6.07 | 4.8 | 7 |
